# Immunogenic Apoptosis as a Novel Tool for Anticancer Vaccine Development

**DOI:** 10.3390/ijms19020594

**Published:** 2018-02-16

**Authors:** Barbara Montico, Annunziata Nigro, Vincenzo Casolaro, Jessica Dal Col

**Affiliations:** 1 Centro di Riferimento Oncologico, Department of Translational Research, Immunopathology and Cancer Biomarkers, 33081 Aviano (PN), Italy; bmontico@cro.it; 2 Department of Medicine, Surgery and Dentistry ”Scuola Medica Salernitana”, University of Salerno, 84081 Baronissi (SA), Italy; anigro@unisa.it (A.N.); vcasolaro@unisa.it (V.C.)

**Keywords:** immunogenic cell death, DAMP, calreticulin, DC-based vaccine

## Abstract

Immunogenic apoptosis, or more appropriately called immunogenic cell death (ICD), is a recently described form of apoptosis induced by a specific set of chemotherapeutic drugs or by physical therapeutic modalities, such as ionizing irradiation and photodynamic therapy. The peculiar characteristic of ICD is the ability to favor recognition and elimination of dying tumor cells by phagocytes in association with the release of pro-inflammatory molecules (such as cytokines and high-mobility group box-1). While in vitro and animal models pointed to ICD as one of the molecular mechanisms mediating the clinical efficacy of some anticancer agents, it is hard to clearly demonstrate its contribution in cancer patients. Clinical evidence suggests that the induction of ICD alone is possibly not sufficient to fully subvert the immunosuppressive tumor microenvironment. However, interesting results from recent studies contemplate the exploitation of ICD for improving the immunogenicity of cancer cells to use them as an antigen cargo in the development of dendritic cell (DC) vaccines. Herein, we discuss the effects of danger signals expressed or released by cancer cells undergoing ICD on the maturation and activation of immature and mature DC, highlighting the potential added value of ICD in adoptive immunotherapy protocols.

## 1. Introduction

Resistance to cell death and the ability to elude immunological surveillance are two of the most malicious strategies allowing the persistence of tumor cells in cancer bearing hosts [1]. Therefore, not only restoring tumor cell susceptibility towards death, but also strengthening and enhancing immune recognition of poorly immunogenic cancer cells would be of high therapeutic value [2]. A particularly attractive perspective, in this respect, is the combination of these two approaches, which would take advantage of the possible immunogenic properties of distinct forms of cancer cell death. In the past, necrosis was the only type of cell death considered to be immunologically harmful, being highly inflammatory because of the sudden release of several intracellular factors, cytokines, and other pro-inflammatory mediators [2]. In contrast, apoptosis, which is a prominent “physiological” pathway of cell death, was considered to be immunologically silent or even tolerogenic. Late apoptotic cells, in fact, are recognized rapidly and specifically by phagocytic cells (e.g., macrophages, immature dendritic cells (DC), endothelial cells, and fibroblasts), owing to surface exposure of various “eat me” signals, with a concomitant suppression of “don’t eat me” signals [3]. Recent studies, however, have challenged this conceptual dichotomy and shed light on crucial interactions between apoptotic tumor cells and some components of the immune system, indicating that an immunogenic type of apoptosis could be induced by a specific set of chemotherapeutic agents (e.g., anthracyclines and mitoxantrone) [4,5] or by physical therapeutic methods (e.g., radiotherapy, hypericin-based photodynamic therapy (Hyp-PDT) and high hydrostatic pressure (HHP)) [6,7]. Compared to “classical” apoptosis, immunogenic apoptosis, more properly called immunogenic cell death (ICD), is characterized by the ability to stimulate the host immune system and to enhance immunological responses to such immunotherapy protocols, as DC-based cancer vaccines [8]. In fact, cancer cells undergoing in vitro drug-induced ICD are capable of mediating an “anticancer vaccine effect” once implanted subcutaneously into immunocompetent mice [4]. Moreover, DC have been shown to play a central role in the recognition of apoptotic cells and in the initiation of an immune response, owing to various cell death-associated stimuli [4,7,9]. One of the main characteristics of immunogenic damaged/dying cells is the exposure on the plasma membrane or secretion of intracellular molecules normally hidden within live cells, which acquire immunostimulatory properties. These molecules belong to the “damage-associated molecular pattern” (DAMP) family and may exert various effects on antigen-presenting cells (APC), including maturation, activation, and enhanced antigen processing/presentation [10,11,12].

In the last decade, several studies have re-evaluated the efficacy of conventional chemotherapeutic agents in the light of their possible abilities to induce ICD, in the attempt to optimize their clinical use and to justify their selection over other, more immunosuppressive drugs [13]. While many data generated in vitro and in animal models (reviewed in [5]) demonstrate the induction of ICD by these agents, clinical and translational evidence in support of these data lag behind. Indeed, cell death-associated anti-tumor immunity has been rarely observed or inferred in cancer patients, presumably due to the inability of chemotherapeutic drugs to induce ICD at concentrations lower than the in vivo maximal tolerable doses. Conversely, encouraging results have been obtained in studies exploring the use of ICD-experiencing cancer cells as sources of tumor antigens in the development of DC-based therapeutic vaccines [8,14,15,16,17,18,19,20]. This strategy allows overcoming several clinical constraints, including the employment of ICD inducers not currently used in cancer management or their use at higher concentrations than those tolerated in vivo. Moreover, the exploitation of ICD ex vivo to develop a DC-based vaccine offers the advantage to markedly boost the host immune system through a tumor-specific and active immunotherapeutic approach. Here we discuss the results of recent studies on the effects of ICD-experiencing tumor cells, and particularly of the ICD-derived DAMP, on the maturation and functional activation of immature (iDC) [21,22,23] and mature DC (mDC) [18,20]. These studies show that tumor cell lysates (TCL) obtained from cells exposed to different ICD inducers could augment the expression of costimulatory molecules and strengthen the T-cell priming ability of mDC generated in vitro with different cytokine cocktails [18,20]. These observations indicate the immunogenic potential of ICD-derived TCL as tumor antigen cargoes in the development of DC-based vaccines.

## 2. ICD Definition and Principal Markers

ICD is a well-defined process characterized by the sequential emission of specific molecules belonging to the DAMP family, normally sequestered within live cells, which may acquire distinct novel functions upon surface exposure or secretion by dying cells. This process is spatially and temporally defined and can be induced by some, but not all, chemo-therapeutic agents. These compounds belong to distinct chemical classes, e.g., doxorubicin, oxaliplatin, mitoxantrone, retinoic acid (RA)/interferon (IFN)-α, and shikonin (SK), and must be cytotoxic in order to attain DAMP exposure/release and the development of an intense immunogenic signal [4,14,20,24,25,26].

DAMP may be subdivided into three major subclasses on the basis of their stage and site of localization/release: (1) DAMP exposed on plasma membrane, e.g., heat-shock protein (HSP)70, HSP90, and calreticulin (CRT); (2) DAMP secreted extracellularly, e.g., high-mobility group box 1 (HMGB1), uric acid, and pro-inflammatory cytokines; and (3) DAMP released as end-stage degradation products, e.g., ATP, DNA, and RNA [2,27,28].

### 2.1. CRT

CRT is a calcium-binding protein, located in the lumen of the endoplasmic reticulum (ER). CRT is mainly involved in the modulation of calcium signaling and homeostasis [29,30], acts as chaperone of several proteins and interplays in particular with the disulfide isomerase Erp57 [31]. CRT acts as a DAMP after exposure (ecto-CRT) on the surface of dying cells in response of specific inducers/stressors in the very early stage of ICD [4]. CRT exposure occurs before the flip of phosphatidylserine on the outer leaflet of the cell membrane. Its mechanism has been thoroughly defined and involves ER stress induction, characterized by the activation of the ER-sessile kinase PERK that phosphorylates and inhibits the eukaryotic translation initiation factor (eiF)-2α [32,33]. Erp57 controls CRT exposure and the two proteins are translocated together in the same molecular complex [34]. Ecto-CRT acts principally as an “eat me signal” delivering the activation of a phagocytic signal to professional APC [4,35]. Erp57 knockdown suppresses CRT exposure and phagocytosis by DC and prevents the development of immunogenicity in vivo [34]. The effect of CRT is counterbalanced by CD47, which inhibits phagocytosis by blocking the recognition and uptake of dying cells by phagocytes via binding to the tyrosine-based inhibitory motif (ITIM)-containing receptor, signal regulatory protein (SIRP) α [36,37,38]. The induction of ecto-CRT expression is usually associated with the concomitant decrease of CD47 levels, favoring the susceptibility of dying cells to engulfment by APC.

### 2.2. HSP70 and HSP90

Like CRT, also HSP70 and HSP90 are exposed on the cell surface during the early stage of ICD, but they are subsequently released in the tumor microenvironment. Inducible HSP are a class of chaperones ensuring the correct folding and subcellular transport of newly synthesized proteins. HSP are also involved in the refolding or degradation of stress-accumulated misfolded proteins [39]. Their translocation from the cytosol to the plasma membrane plays a dual role in cancer cells [2]. Intracellular overexpression of HSP70 and HSP90 favors cancer cell survival by apoptosis inhibition [39]; conversely, exposed HSP suppress tumor survival by attracting innate immune system cells [35]. In cancer cells, both HSP70 and HSP90 directly interact with apoptosis protease-activating factor-1 (Apaf-1), thereby inhibiting the formation of the apoptosome via blocking the recruitment of procaspase-9 [40,41]. When exposed, these chaperones are responsible for cancer cell-immune cell interaction by binding Toll-like receptors (TLR) on DC and NKG2A on natural killer (NK) cells [11]. In line with these notions, high levels of HSP70 and HSP90 have been detected in the serum of cancer patients in the presence of such stress conditions, as inflammation, bacterial and viral infections. [42]. Intriguingly, as shown by Lin and coworkers in mice vaccinated with shikonin (SK)-treated TCL-loaded DC, HSP70 appeared to further boost tumor-directed immunity by inducing a significant decrease of circulating CD11b^+^Ly6C^+^ monocytic and CD11b^+^Ly6G^+^ granulocytic myeloid-derived suppressor cells (MDSC) [43].

### 2.3. HMGB1

HMGB1 is a non-histone chromatin-binding protein universally expressed by nucleated cells. Nuclear HMGB1 participates in transcriptional regulation acting as a DNA chaperone with DNA binding and bending activities [44]. Although HMGB1 is normally located in the nucleus, it can translocate from the nucleus to the cytosol following exposure to various stressors, where it mainly acts as a positive regulator of cytoprotective autophagy processes [45,46,47,48]. Moreover, HMGB1 can be passively released in the extracellular environment by necrotic cells [12], where it plays a critical role in activating the inflammatory process. Like CRT and HSP species, this protein also acts as a DAMP in the context of ICD [49]. However, since HMGB1 release requires injured nuclear and plasma membranes, its exposure occurs during the late stages of ICD, hence occurring well after CRT and HSP exposure. Once released from dying cells, HMGB1 functions as a pro-inflammatory cytokine binding to different receptors on DC and promoting antigen presentation.

### 2.4. ATP

The release of ATP by dying tumor cells is another key aspect of ICD [50]. Secretion of cytoplasmic ATP by dying cancer cells requires the activation of multiple signal transduction pathways and could involve gap junction hemi-channels and pannexin channels among others. In particular, the triggering of autophagy, in combination with caspase activation, is needed for ATP secretion from ICD-experiencing cells. Hence, inhibition of autophagy, which limits the release of ATP in response to chemotherapy, results in decreased recruitment of immune effector cells [51,52]. ATP secretion during ICD requires the relocalization of ATP from lysosomes to autolysosomes together with the redistribution of lysosomal-associated membrane protein 1 (LAMP1) to the plasma membrane [53]. The involvement of caspase activation does not appear to be necessary for all ICD inducers. In the case of Hyp-PDT, secretion of ATP occurs through the activation of PERK- and phosphoinositide 3-kinase (PI3K)-mediated mechanisms and independently of caspase signaling [54]. ATP released by dying tumor cells functions as a “find me” signal attracting myeloid cells, including DC, to the tumor bed via binding to the purinergic receptor, P2X7, on DC and stimulating NLRP3 inflammasome activation and the proteolytic maturation of IL-1β [55].

Another molecule which could recruit tumor-infiltrating T lymphocytes upon induction of ICD is the chemokine, C-X-C motif ligand 10 (CXCL10) [56]. Anthracyclin, mitoxantrone and other ICD-inducing chemotherapeutic agents may promote the secretion of CXCL10 upon triggering type I IFN signaling in cells undergoing ICD, CXCL10 production requires the activation of the endosomal pattern recognition receptor TLR3 through the binding of RNA molecules released from dead cells. In turn, by binding to CXC-chemokine receptor 3 (CXCR3) in autocrine and paracrine circuitries, secreted CXCL10 influences the susceptibility of cancer cells to ICD [19]. Furthermore, CXCL10 could exert its chemokine functions and attract immune cells to the tumor microenvironment contributing directly to ICD-elicited immune responses.

## 3. The Critical Role of CRT in Regulating ICD-Dependent Immune Responses

Ecto-CRT is recognized as the earliest and the most important marker of ICD. Obeid and collaborators demonstrated for the first time that ecto-CRT is involved in anthracyclin- and mitoxantrone-induced apoptosis and that there is a strong, positive linear correlation between the exposure of CRT and the immunogenicity elicited by distinct apoptosis inducers [4]. The knockdown of CRT by specific siRNA or its blockade by specific antibody compromised the immunogenicity of mitoxantrone-treated CT26 mouse colon cancer cells, which was restored when recombinant CRT was used to compensate for the CRT defect. Moreover, in vivo experiments showed that dying CT26 cells exposed to ICD inducers and then injected into one flank of immunocompetent BALB/c mice protected the animals against tumor growth when they were subsequently rechallenged with live tumor cells injected into the opposite flank [4,13]. This protective function was lost when experiments were conducted in athymic (nu/nu) BALB/c mice, indicating that tumor growth was inhibited by active immune responses [4,13]. Recently, Pozzi et al. confirmed that CRT exposure, together with ER-stress induction, is critical for immunogenicity of apoptotic cancer cells, as they showed that human colon cancer cells that did not expose CRT after cetuximab treatment escaped recognition and phagocytosis by DC [57].

Accordingly, elevated CRT expression levels on tumor cells in non small cell lung carcinoma patients correlate with the presence of infiltrating mDC and effector memory T cells [58]. In addition a bioinformatic study showed the impact of CRT mRNA expression levels in tumor cells on the density and composition of immune infiltrates. In particular, CRT expression correlates with DC and cytotoxic T lymphocyte (CTL) infiltration in breast, colon, and ovarian cancers suggesting that the loss of CRT expression may negatively affect immunosurveillance [59]. The potential contribution of human blood DC in mediating immune responses induced by ICD was documented by Di Blasio et al. that showed the ability of these DC to engulf tumor cells previously treated with platinum compounds. Moreover, the phagocytosis of treated tumor fragment increased phenotypic maturation of blood myeloid and plasmocytoid DC [60]. Although in most works studying ICD the attention is focused on DC, it has been demonstrated that the induction of ecto-CRT and its release in the surrounding milieu after UVB irradiation promoted tumor fragment endocytosis by macrophages. Moreover, the internalization of exogenous CRT by macrophages resulted in phenotypic changes such as an increase of cell spreading and migration, an upregulation of CD14, an increase of interleukin-8 release [61]. Duo and collaborators showed the ability of soluble CRT to promote pro-inflammatory phenotype in macrophages through the activation of MAPK and NF-κB pathways resulting in TNF-α and IL-6 production [62]. Therefore, further studies investigating the potential involvement of other immune interactors in ICD-induced immune responses may describe a more complex landscape.

However, the key role of ecto-CRT was not consistently appreciated when TCL from cells undergoing ICD were used for DC loading in vaccine development protocols [19,20,43]. It appears then that the impact of CRT in TCL immunogenicity may vary widely depending on the ICD inducer. CRT blockade with a specific antibody during DC loading with TCL derived from mantle cell lymphoma cells (Mino) undergoing ICD upon RA/IFN-α treatment significantly reduced the killing activity of tumor-specific CTL generated by in vitro co-culture with ICD-TCL-pulsed DC [20]. Conversely, ecto-CRT blockade during DC pulsing with TCL from murine glioma cells (GL261) previously exposed to Hyp-PDT slowly, and non-significantly attenuated the immunogenic potential of the ICD-DC-based vaccine in vivo [19]. Furthermore, upregulated CRT induced by SK in mouse mammary carcinoma cells (4 T1) stimulates the proliferation of CD8^+^, but not of CD4^+^, lymphocytes in mice vaccinated with SK-TCL pulsed DC [43]. Moreover, SK-induced HSP70 and HMGB1, but no CRT, influence the secretion of cytokines and chemokines by TCL-loaded mature DC [43].

## 4. The Immunostimulatory Potential of ICD-DAMP on Immature and Mature DC

**Figure 1:**
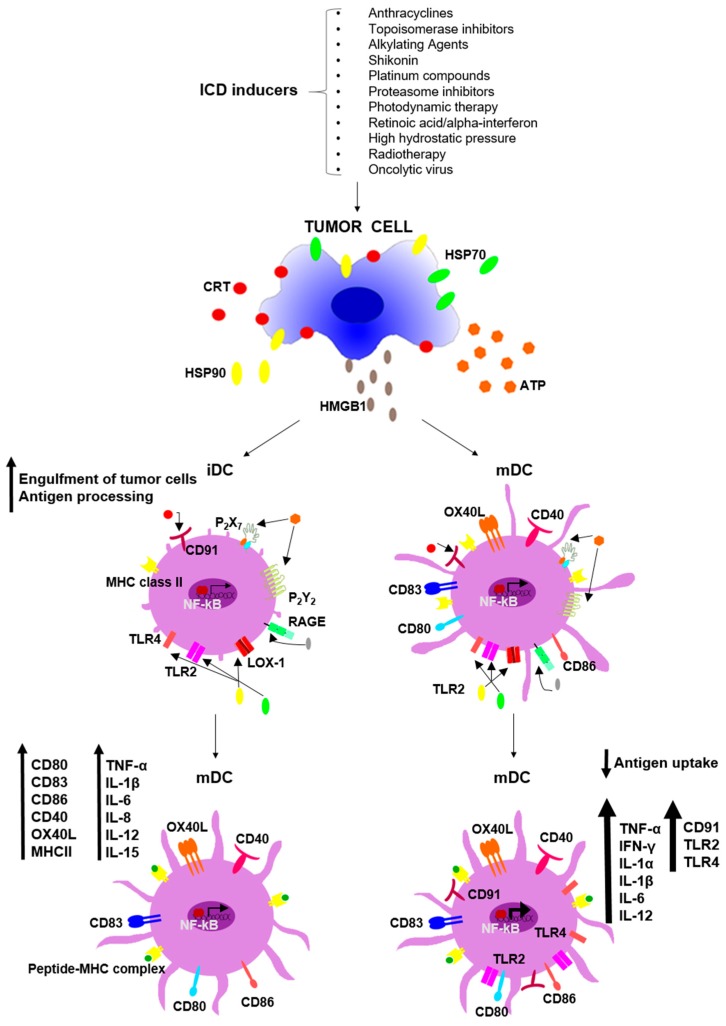
Schematic illustration of ICD-induced damage-associated molecular pattern (DAMP) impact on iDC and mDC. The exposure on the cell surface of CRT, HSP70 and HSP90 favors the engulfment of dying cancer cells by iDC (on the left). The chaperones released in the extracellular environment, together with high-mobility group box 1 (HMGB1) and ATP, promote the maturation of iDC characterized by expression of several surface markers and the release of pro-inflammatory cytokines. Similarly, the released DAMP can bind specific receptors on mDC (on the right), enhancing their activation and their ability to stimulate T-cell mediated immune responses.

The immunogenic potential of ICD derives from the immunostimulatory properties of the peculiar DAMP that characterize this form of apoptosis. Specifically, the molecules secreted by dying cells during the late stages of ICD are able to directly bind and activate DC (Figure 1 and Table 1) or to attract immune cells into the tumor microenvironment. Functional activation of DC is associated with a maturation process characterized by a wide variety of cellular changes, including (1) development of dendrites; (2) decreased antigen capture; (3) increased expression of surface MHC class I and II molecules; (4) upregulated expression of costimulatory molecules; (5) acquisition of chemokine receptors; and (6) enhanced production and secretion of cytokines and chemokines that control T-cell differentiation and recruitment (reviewed in [63]). DC maturation and activation can be promoted by the ICD-DAMP through the interaction with a host of specific receptors [54,55,64,65] and the resulting activation of NF-κB [66], IFN-regulatory factor (IRF) [20,56], and the MAPK and Akt pathways (JDC, unpublished data). Whereas the function of MAPK molecular species and of Akt kinase in the regulation of DC biology and activity has yet to be fully elucidated [67,68,69], the key role of NF-κB signaling is consistently documented in a number of studies [70,71,72,73].

### 4.1. DAMP Effects on iDC

Ecto-CRT binds to several receptors present on APC, including thrombospondin, complement component 1, q subcomponent (C1q) receptors, mannose-binding lectin and CD91 (LDL-receptor-related protein or LRP1), which stimulates Rac-1 and promotes the engulfment of apoptotic cells. The axis formed by ecto-CRT and CD91 is particularly critical; in fact, a blockade of ecto-CRT decreases the immunogenicity of anthracycline-induced ICD in vitro and in vivo, which is restored by addition of a recombinant form of CRT [4]. Moreover, the downregulation of CD91 decreased the immunogenicity of cancer cell death induced by Hyp-PDT [65]. Besides functioning as an “eat me” signal, in the late stage of ICD CRT is secreted in the extracellular milieu where it becomes the soluble ligand for CD91 expressed on DC, leading to its phosphorylation and eventually resulting in NF-κB activation [66]. This in turn induces the production and subsequent release of inflammatory cytokines (particularly IL-1β, IL-12, IL-6 and TNF-α) and chemokines (such as CXCL10 and CXCL11) [66].

Both soluble and membrane-anchored forms of HSP70 and HSP90 can contribute to DC phenotypical and functional maturation through the interaction with CD91, but also with TLR2, TLR4, and lectin-type oxidized LDL receptor 1 (LOX-1) [64,65,74,75]. The signaling cascades primed by all these receptors converge on the activation of the NF-κB and IRF pathways and the ensuing production of pro-inflammatory cytokines [76]. Furthermore, ICD-induced HSP70 and HSP90 upregulate the expression of maturation markers, CD80, CD83, CD86, and MHC class II molecules on iDC, favoring their stimulatory activity towards tumor-specific CD4^+^ and CD8^+^ T cells [21,22,23]. The immunogenic potential of HSP is well-known, as witnessed by the widespread use of heat shock-enriched TCL in DC-vaccine development. Indeed, several studies demonstrated that these preparations induce more pronounced and efficient immune responses relative to untreated TCL [77]. Finally, in damaged or tumor cells, these chaperones are able to capture cellular antigens, including tumor antigens, and facilitate their presentation following uptake by iDC [9].

HMGB1, once released from dying tumor cells, also impacts on antigen processing and presentation by DC. HMGB1 does so via binding TLR2, TLR4, and the receptor for advanced glycosylation end-products (RAGE) expressed on immune cells [64]. Particularly, TLR4 and its adaptor, myeloid differentiation primary response gene 88 (MyD88), were found to be essential for optimal antigen processing by inhibiting the fusion between phagosomes and lysosomes, thereby preventing the degradation of tumor antigens [78,79]. Both TLR4, expressed on APC, and HMGB1 are required for ICD and for efficient immune stimulation as demonstrated in experiments neutralizing HMGB1 [80] and in breast cancer patients having a loss-of-function TLR4 allele exhibiting reduced binding affinity for HMGB1. These patients respond poorly to radiotherapy and anthracycline therapy [49,64]. Thus, the HMGB1/TLR4 axis seems to play a significant role in cancer therapy especially when ICD is one of the mechanisms accounting for the cytotoxic activity of chemotherapeutic agents. When administered to TLR4-expressing DC in vitro, HMGB1 augments the expression of pro-IL-1β and prevents the lysosomal degradation of engulfed tumor antigens, which is a major prerequisite for efficient cross-presentation. HMGB1 can also induce inflammation by inducing the release of such pro-inflammatory cytokines, as TNF-α, IL-6 and IL-8, from myeloid cell populations [81].

Following exposure to ICD inducers, ATP secretion occurs during phosphatidylserine exposure on the cell membrane and involves the activation of autophagy processes [53]. ATP is a potent “find me” signal and can recruit myeloid cells, such as DC, in the intra- and peri-tumoral areas. Moreover, ATP binds the P2Y2 and P2X7 purinergic receptors, resulting in the activation of the NLRP3 inflammasome, promotion of caspase 1 proteolytic activity and production of the pro-inflammatory cytokine, IL-1β [55]. Finally, the release of IL-1β by DC is critical for eliciting tumor-specific IL-17-producing γδ T cells and IFN-γ-producing CTL [82].

### 4.2. DAMP Effects on mDC

The immunostimulatory effects of TCL from cells experiencing ICD have also been demonstrated on mDC. This is particularly attractive in the development of cancer DC-based vaccines, as these would require the loading of immunogenic ICD-TCL to in vitro generated mDC.

The most marked effect of ICD-TCL and DAMP on mDC is the promotion of cytokine and chemokine secretion. SK-TCL-loaded DC, as compared to naïve TCL-loaded DC, release substantially higher levels of chemokines, including CXCL12, CCL2, and CCL3, or macrophage inflammatory protein (MIP)-1α, and of granulocyte-colony stimulating factor (G-CSF), [43]. Different ICD-DAMP affect the production of these molecules. Specifically, HSP-70 increases the secretion of G-CSF, CCL2, and MIP-1α, HMGB1 enhances the levels of CXCL12 and MIP-1α, while CRT promotes a slow increment of G-CSF [43]. Apoptotic murine cell lines, TC-1 and TRAMP-C2, exposed to HHP and used as tumor antigen formulations for pulsing mDC, significantly increased the amounts of IL-12 and IFN-γ secreted by DC compared to unpulsed cells [18] without modifying the expression levels of DC maturation markers. Similarly, TCL from RA/IFN-α-treated lymphoma cells increased the percentage of DC with nuclear NF-κB and the release of IL-1β, IL-1α, IL-6, and TNF-α relative to unpulsed- and naïve TCL-pulsed DC [20]. In the latter study, RA/IFN-α-TCL inhibited antigen uptake by mDC consistent with the acquisition of a more mature phenotype, albeit several maturation markers were not expressed differently in unpulsed and ICD-TCL-pulsed mDC.

Finally, as shown by Lin and collaborators, both HSP70 and HMGB1 upregulated the expression of key ICD molecule-associated receptors, including CD91, TLR2, and TLR4, on mDC. This suggests that certain ICD markers can enhance the responsiveness of DC to ICD-DAMP exposure [43].

## 5. Exploitation of Tumor Cells Undergoing ICD in DC-Based Immunotherapy

Considering that ICD inducers and ICD-induced DAMP not only increase tumor cell immunogenicity, but also enhance DC maturation and DC ability to stimulate immune effector cells, ICD could represent a novel tool for improving DC-based immunotherapy. The first findings supporting this idea were documented by Strome et al. who showed that γ-irradiated (irr)-tumor cell-pulsed DC efficiently stimulate antigen-specific CTL responses [83]. In addition, in mice vaccinated with γ-irr-tumor cell-pulsed DC significant tumor growth suppression was detected following inoculation with live syngeneic tumor cells [83,84]. The use of γ-irr cancer cells in DC vaccine development was successfully implemented in clinical practice in melanoma and high-grade glioblastoma patients resulting in prolonged median overall survival [85]. In addition, Zappasodi and collaborators demonstrated that vaccination with DC loaded with apoptotic autologous tumor cells, which were subjected to exposure to a combined treatment including heat shock, γ-ray and UV ray, could stimulate clinical responses in 33% of relapsed B-cell lymphoma patients [8]. Notably, the authors reported a direct correlation between the levels of ecto-CRT and exposed HSP90 in the tumor cell cargo loaded on DC and the clinical and immunological responses observed in these patients. Intriguingly, the neoplastic cells of non-responder patients were not susceptible to treatment-induced ICD [8].

Promising results in preclinical settings were also obtained in multiple tumor models following HHP treatment. The immunization of immunocompetent mice with DC pulsed with HHP-treated tumor cells induced powerful immune responses, detected by increased spleen cell cytotoxicity and elevated IFN-γ production. HHP-tumor cell-loaded DC administered in combination with docetaxel chemotherapy significantly inhibited the growth of ovarian (TC-1) and prostate (TRAMP-C2) tumors [18]. Promising results were also obtained in the clinical setting of minimal residual tumor disease after surgery, where DC vaccination was shown to reduce the growth of tumor recurrences after surgery of poorly immunogenic transplanted TRAMP-C2 and of immunogenic TC-1 tumors [17].

PDT is another well described physical inducer of ICD. Several studies reported that PDT-induced ICD leads to efficient rejection of murine tumors in vivo in both prophylactic and curative vaccination models [54,86,87]. The intratumoral administration of iDC in combination with PDT treatment resulted in effective DC homing to regional and peripheral lymph nodes. This was associated with an effective tumor eradication subsequent to the stimulation of cytotoxic activity of T and NK cells [88]. DC loaded with PDT-TCL could also increase CTL responses and Th1-driven immunity in established solid non-orthotopic tumors [89] and in orthotopic HGG mouse models [19] leading to tumor growth inhibition and survival benefit. Notably, Hyp-PDT-TCL-loaded DC vaccines also induced an immunostimulatory shift in the brain immune environment, leading to reduced numbers of regulatory T cells and increased prevalence of Th1, CTLs, and Th17 lymphocytes [19].

Mice immunized with DC loaded with SK-TCL obtained from B16 melanoma cells and subsequently inoculated with live B16 cells showed a significant delay in tumor growth associated with increased tumor-specific cytolysis mediated by splenocytes [43]. Recently, we optimized a novel strategy to induce ICD in B-lymphoma cells through the combination of RA plus IFN-α [20]. Using this approach, we showed that RA/IFN-α-TCL activated mDC and made them more efficient in eliciting tumor antigen-specific CTL in vitro, by stimulating Th1 and Th17 cells, relative to DC unpulsed or pulsed with naïve-TCL. The in vivo therapeutic potential of RA/IFN-α-TCL pulsed-DC vaccine was assessed in immunodeficient NOD/SCID mice reconstituted with human peripheral blood lymphocytes, where this vaccine inhibited lymphoma growth. The high levels of IFN-γ detected in the sera of treated mice is consistent with the ability of RA/IFN-α-TCL pulsed-DC to induce a systemic Th1-skewed immune response [16].

Oncolytic viruses (OV) are another promising class of immunotherapy agents. Besides selectively and directly lysing infected tumor cells, OV elicit a potent antitumor immune response. OV replicate in cancer cells and release tumor antigens, which are perceived as dangerous because of the simultaneous expression of pathogen-associated molecular patterns that activate APC. At least three OV, coxsackievirus B3, oncolytic adenovirus, and Newcastle disease virus, have proved effective at inducing ICD [15,90,91].TCL obtained from OV-infected tumor cells, called oncolysates, could provide a tumor antigen cargo for DC. The superior effectiveness of OV-TCL-pulsed DC vs naïve-TCL-pulsed DC in stimulating tumor-specific CTL responses in vitro has been convincingly demonstrated, but no preclinical in vivo data are available yet confirming the efficacy of oncolysate-loaded-DC. However, different studies have documented the beneficial effects of intratumoral administration of OV in combination with tumor-directed systemic DC vaccination [92,93].

## 6. Conclusions

DC-based immunotherapy shows the advantage to induce an adaptive immune response against the tumor, with the potential to generate a long-lasting immunological memory able to prevent further relapses and hopefully metastasis. While promising, the clinical benefit provided by this immunotherapeutic approach is still limited and the choice of the optimal antigen formulation remains one of the unresolved issues in DC-vaccine protocol development. The employment of TCL as a source of antigens to pulse DC is a strategy adopted in several clinical trials that administer autologous DC vaccines. The main advantages of using TCL are the opportunity of loading a wide variety of antigens and the capacity to induce both CD4^+^ and CD8^+^ T-cell responses. To improve the clinical efficacy of a DC-based vaccine, different treatment methods were developed for enhancing the immunogenic potential of tumor cell cargoes generated for DC loading. Although only few of all described ICD inducers have been evaluated for their ability to generate an immunogenic cell cargo to load DC vaccines, results to date have been obtained in multiple preclinical tumor models.

One of the major advantages of this strategy is that, under these conditions, DC are not only exposed to tumor-associated antigens but also to DAMP that can affect DC biology improving their immunological activities. On the other hand, it is unlikely that the induction of ICD alone can fully subvert the immunosuppressive tumor microenvironment. Therefore, combining the immunogenic potential of ICD with an active immunotherapeutic protocol such as DC vaccination is a promising approach which could prove clinically successful in future studies.

## Figures and Tables

**Table 1:** Main immunogenic cell death (ICD)-inducers and their effects on immature dendritic cells (iDCs) and mature dendritic cells (mDCs).

ICD Inducer	ON-Target Effect	DAMPs	Effects on iDCs	Effects on mDCs
Anticancer drugs inducing ICD				
Anthracyclines (Doxorubicin, Epirubicin, Idarubicin)	DNA damage	Exposure of CRT and HSP70; Release of HMGB1; Secretion of ATP	Increased tumor cell engulfment and antigen processing; Induced DC maturation: ◦ increased expression of CD40; ◦ release of IL-1β, Il-6 and IL-12	Enhanced expression of CD80/CD86 and CD83; Release of IL-1β
Topoisomerase inhibitors (Mitoxantrone)	Intercalating agent	Exposure of CRT and HSP70	Increased antigen processing and cross-presentation; induced DC maturation: ◦ release of IL-12, IL-6, IL-23 and TGF-β ◦ release of IL-1β, IL-6, IL-12 e TNF-α	
Alkylating agents (Cyclophosphamide)	DNA alkylation	Exposure of CRT; Release of HMGB1; Secretion of ATP	Increased antigen processing and cross-presentation	
Shikonin	DNA damage	Exposure of CRT and HSP70	Induced DC maturation: ◦ increased expression of CD80/CD86 and CD40; ◦ release of IL-12, IL-6, IL-23 and TGF-β	Release of CXCL12 and CCL3; Increased expression of CD91, TLR2 and TLR4.
Platinum Compounds (Oxaliplatin)	DNA damage	Exposure of CRT and HSP70	Increased antigen processing and cross-presentation	Release of IL-12
Proteasome inhibitor (Bortezomib)	Proteasomal inhibitor	Exposure of CRT, HSP70 and HSP90; Release of HMGB1	Increased antigen processing and cross-presentation	
Retinoic acid/alpha-interferon	Aspecific inhibition of PI3-k/Akt pathway	Exposure of CRT, HSP70 and HSP90; Release of HMGB1	Increased phagocytosis	Reduced antigen uptake; NF-kB signaling activation; release of IL-1β, IL-1α, IL-6 and TNF-α
Physical modalities inducing ICD					
High hydrostatic pressure	Membranes disruption and protein denaturation	Exposure of CRT; Release of HSP70, HSP90 and HMGB1; Secretion of ATP	Increased phagocytosis; induced DC maturation: ◦ increased expression of CD80/CD86 and CD83	Release of IL-12 and INF-γ
Radiotherapy	DNA damage	Exposure of CRT and HSP70; Release of HMGB1	Increased phagocytosis; Induced DC maturation	Release of IL-6
Photodinamic Therapy	Reactive Oxygen Species mediated ER membrane damage	Exposure of CRT; Release of HSP70, HSP90 and HMGB1; Secretion of ATP	Increased phagocytosis; Induced DC maturation: ◦ increased of CD80/CD86, CD83; ◦ enhanced of MHC-II	Release of IL-1β, IL-6 and IL-12
Oncolytic virus	Lysis of tumor cells through ER damage	Exposure of CRT; Release of HMGB1; Secretion of ATP	Induced DC maturation: ◦ increased expression of CD80/CD86

## Abbreviations

Apaf-1	apoptosis protease-activating factor-1
APC	Antigen presenting cell
CRT	Calreticulin
CTL	Cytotoxic T lymphocyte
CXCL	C-X-C motif ligand
CXCR	C-X-C chemokine receptor
DAMP	Damage associated molecular pattern
DC	Dendritic cell
eiF2-α	eukaryotic translation initiation factor 2-α
ER	Endoplasmic reticulum
HHP	High hydrostatic pressure
HMGB1	High mobility group box 1
HSP	Heat shock protein
Hyp-PDT	Hypericin-photodynamic therapy
ICD	Immunogenic cell death
iDC	Immature DC
IFN	Interferon
IL	Interleukin
IRF	Interferon regulatory factor
irr	Irradiation
ITIM	tyrosine-based inhibitory motif
LAMP1	Lysosomal-associated membrane protein 1
mDC	mature DC
MDSC	myeloid derived suppressor cell
NK	natural killer
OV	oncolytic virus
PERK	protein kinase RNA-like endoplasmic reticulum kinase
PI3K	phosphoinositide 3 kinase
RA	retinoic acid
SIRPα	Signal regulatory protein α
SK	shikonin
TCL	tumor cell lysate
TLR	toll-like receptor
